# The Communicating Narrative Concerns Entered by Registered Nurses (CONCERN) Clinical Decision Support Early Warning System: Protocol for a Cluster Randomized Pragmatic Clinical Trial

**DOI:** 10.2196/30238

**Published:** 2021-12-10

**Authors:** Sarah Collins Rossetti, Patricia C Dykes, Christopher Knaplund, Min-Jeoung Kang, Kumiko Schnock, Jose Pedro Garcia Jr, Li-Heng Fu, Frank Chang, Tien Thai, Matthew Fred, Tom Z Korach, Li Zhou, Jeffrey G Klann, David Albers, Jessica Schwartz, Graham Lowenthal, Haomiao Jia, Fang Liu, Kenrick Cato

**Affiliations:** 1 Department of Biomedical Informatics Columbia University New York, NY United States; 2 School of Nursing Columbia University Medical Center New York, NY United States; 3 Brigham and Women's Hospital Boston, MA United States; 4 Harvard Medical School Boston, MA United States; 5 Working Diagnosis Haddonfield, NJ United States; 6 Anschutz Medical Campus University of Colorado Aurora, CO United States

**Keywords:** nursing documentation, prediction, early warning system, deterioration, clinical trial, clinical decision support system, natural language processing

## Abstract

**Background:**

Every year, hundreds of thousands of inpatients die from cardiac arrest and sepsis, which could be avoided if those patients’ risk for deterioration were detected and timely interventions were initiated. Thus, a system is needed to convert real-time, raw patient data into consumable information that clinicians can utilize to identify patients at risk of deterioration and thus prevent mortality and improve patient health outcomes. The overarching goal of the COmmunicating Narrative Concerns Entered by Registered Nurses (CONCERN) study is to implement and evaluate an early warning score system that provides clinical decision support (CDS) in electronic health record systems. With a combination of machine learning and natural language processing, the CONCERN CDS utilizes nursing documentation patterns as indicators of nurses’ increased surveillance to predict when patients are at the risk of clinical deterioration.

**Objective:**

The objective of this cluster randomized pragmatic clinical trial is to evaluate the effectiveness and usability of the CONCERN CDS system at 2 different study sites. The specific aim is to decrease hospitalized patients’ negative health outcomes (in-hospital mortality, length of stay, cardiac arrest, unanticipated intensive care unit transfers, and 30-day hospital readmission rates).

**Methods:**

A multiple time-series intervention consisting of 3 phases will be performed through a 1-year period during the cluster randomized pragmatic clinical trial. Phase 1 evaluates the adoption of our algorithm through pilot and trial testing, phase 2 activates optimized versions of the CONCERN CDS based on experience from phase 1, and phase 3 will be a silent release mode where no CDS is viewable to the end user. The intervention deals with a series of processes from system release to evaluation. The system release includes CONCERN CDS implementation and user training. Then, a mixed methods approach will be used with end users to assess the system and clinician perspectives.

**Results:**

Data collection and analysis are expected to conclude by August 2022. Based on our previous work on CONCERN, we expect the system to have a positive impact on the mortality rate and length of stay.

**Conclusions:**

The CONCERN CDS will increase team-based situational awareness and shared understanding of patients predicted to be at risk for clinical deterioration in need of intervention to prevent mortality and associated harm.

**Trial Registration:**

ClinicalTrials.gov NCT03911687; https://clinicaltrials.gov/ct2/show/NCT03911687

**International Registered Report Identifier (IRRID):**

DERR1-10.2196/30238

## Introduction

### Background

Every year, more than 330,000 inpatient deaths resulting from cardiac arrest and sepsis occur [1,2], which could otherwise have been avoided if those patients’ risk for deterioration was detected earlier and meaningful interventions were provided. While clinicians strive to provide the best quality care, suboptimal interprofessional communication between nurses and physicians can lead to delays in patient care [3-6]. Nurses often observe subtle yet concerning changes in their patients even before physiological conditions start to deteriorate. When they do, they tend to increase surveillance among those patients and documentation within the electronic health record (EHR) [3,7-9]. However, owing to systemic problems within hospital settings, such as physicians frequently needing to round between multiple units or not having substantial time to review nurses’ documentation, physicians and nurses may have different understandings of a patient’s situation. A lack of shared situational awareness can inhibit the care team’s ability to deploy early interventions directed at deterioration prevention, placing patients at greater risk for deterioration. Real-time processing and conversion of raw patient data into consumable information, displayed using “smart” visualizations, are therefore needed within EHRs to ameliorate these deficiencies and ensure that hospitalized patients at high risk for deterioration are rapidly identified with equal understanding by all members of the care team.

Implementing an early warning system (EWS) within EHRs would provide users with a tool to communicate nurses’ emerging concerns about changes in patient states to the interprofessional team. EWSs have assisted clinicians in detecting patients’ risk of deterioration since the 1990s [10]. In the past, EWS algorithms were often built using clinicians’ consensus [11]. Recently, as we reported previously, approaches to EWS algorithms have shifted to become more data-driven [11], which can reveal hidden relationships that are difficult to detect by humans. Today’s EWS algorithms, therefore, more commonly leverage vast numbers of variables available in the EHR, such as vital signs, level of consciousness, and laboratory data.

The COmmunicating Narrative Concerns Entered by RNs (CONCERN) study is developing and evaluating the impact of a new EWS that predicts and provides clinical decision support (CDS) when patients are at increased risk of deterioration. Compared to existing EWSs, the CONCERN CDS system defined a new source of predictive data, analyzing nursing documentation patterns that reflect nursing surveillance and indicate nurses’ changing levels of concern. Our preliminary study using the aforementioned approach has demonstrated that using nursing documentation patterns as an EWS predictor performed similarly to the Modified Early Warning Score (MEWS), one of the most widely used EWSs in clinical settings, which leverages the actual recorded values (eg, a heart rate of 60 BPM) as its predictors [12]. However, CONCERN was able to detect patient deterioration 42 hours earlier than the MEWS [13]. CONCERN’s earlier detection capabilities therefore create a more advantageous window of opportunity for clinicians to anticipate and appropriately react to impending patient deterioration.

This study proposes the CONCERN intervention trial design, a multiple time-series intervention evaluating the system’s implementation through efficacy evaluation to understand how the CONCERN CDS system performs in the clinical setting.

### Study Objectives

#### Primary Objective

This multi-site cluster randomized pragmatic clinical trial study [14] (between New York Presbyterian and Mass General Brigham) will assess quantitative CDS usage and monitoring data to evaluate the effectiveness of implementing the CONCERN CDS system to decrease hospitalized patients’ negative health outcomes on acute and critical care units.

#### Secondary Objectives

This study’s secondary objectives are to evaluate qualitative CONCERN CDS system usage and to conduct usability surveys focused on the following topics: (1) perceived understandability of the CONCERN CDS app, (2) perceived technical competence of the CONCERN CDS app, and (3) trust in the CONCERN CDS app (as influenced by understandability and technical competence).

### CONCERN CDS System Development

The CONCERN CDS system will be triggered on the basis of analytics of nursing documentation through NLP, which indicates the recognition of and concerns about negative patient changes [13,15]. Testing of the predictive model underlying the CONCERN CDS has been conducted on retrospective data and findings have been published, including those that previously highlighted that CONCERN performs similarly to the MEWS with an improved lead time of 42 hours [13,15]. The CONCERN CDS app will alert the interprofessional care team to the patients entering “risky states” to increase team-based situational awareness of these patients and support them as they perform early clinical interventions.

#### Conceptual Framework

A Healthcare Process Modeling Framework to Phenotype Clinician Behaviors for Exploiting the Signal Gain of Clinical Expertise (HPM-ExpertSignals) was used as the fundamental conceptual framework of the CONCERN model [13]. This model identifies features from user interaction with clinical systems, which are patterns of clinical behaviors that can be interpreted as proxies of individuals’ decisions, knowledge, and expertise. These proxies, in turn, can be used in predictive models to identify associations with outcomes. In developing the CONCERN early warning score, increased surveillance beyond the standard of care was used as an indicator of acute concern about patient deterioration.

#### Development: CONCERN CDS Scoring Engine

The development of the CONCERN CDS scoring engine was separated into three stages: (1) feature selection and preprocessing, (2) feature modeling, and (3) assignment of colors and postprocessing. Expert consensus, clinician feedback, and evaluation of machine learning performance were used in each stage to develop and assess the engine. Initial features selected from prior research [7], vital signs and vital sign comments frequency, were combined with features selected by experts and were thought to be signals of clinical concern. Additional added features including pro re nata (PRN) medications that were administered, medications of any type that were withheld, frequency of nursing notes being written, and nursing note content were also included in the model, along with the times that those actions were performed [13]. Features were iteratively aggregated over the past 12 hours. Final features used in the algorithm are informed by our cumulative qualitative and quantitative analyses over the years. These features were combined using machine learning techniques (NLP, decision trees, and logistic regression) with proxies for clinical deterioration such as rapid response, cardiac arrest, sepsis, unanticipated intensive care unit (ICU) transfer, and death as the outcomes. A logistic regression–based model was chosen for implementation because focus group with clinicians indicated that model explainability was important. In addition, based on the existing information technology (IT) infrastructure at Mass General Brigham’s (MGB) hospital, logistic regression models were the most feasible to implement. The weights derived from machine learning were used to combine the features into a single score that was reflective of clinical concern for patient deterioration. Color coding of the score and other postprocessing was carried out to incorporate feedback from clinicians, adjust for demographic disparities, and retain the signals obtained from machine learning. We specifically evaluated our model to identify and mitigate any racial disparities and have presented those results here [13,15]. The resulting CONCERN score is a color-coded score representative of changes in surveillance patterns indicative of a patient’s degree of risk for deterioration. A red score signifies a high CONCERN level, which implies that the patient is actively showing signs of deterioration. A yellow score is arguably the most important, which implies that the patient is at increased risk for deterioration, but not yet showing signs of active deterioration. A green score represents a low CONCERN level, which implies that the patient is at low risk for deterioration. All patients will have a CONCERN level of gray (nonscore) until they have 12 hours of history under acute care. To minimize alert fatigue and based on our analysis of historical data, we configured the predictive model to targeted specific percentiles of patients in the units with the following scores: 2%-3% are red and 20%-25% are yellow.

#### Development: Iterative, Participatory Approach to Design the CONCERN CDS App

A multi-method approach, consisting of user-centered design sessions, focus group interviews, and simulation testing sessions with nurses and physicians, was used to facilitate participatory design of our CONCERN CDS app. The design was updated iteratively after each stage on the basis of feedback collected from the participants.

User-centered design sessions were conducted to evaluate the preliminary design of the CONCERN CDS app. Participants were probed about features of CDS tools they used in their practice, which they perceived as useful. Then, focus groups were conducted, during which the results of the preliminary data analysis were presented in addition to mockups of the front-end design of the CONCERN CDS app integrated into the EHR system. Clinicians were asked to provide their opinions on the clinical significance of our findings and their perceptions of the front-end design. The last stage of the participatory process was simulation testing. We used a web-based, functional CONCERN CDS prototype to assess the tool’s usability and functionality within existing end user workflow.

#### Development: CONCERN CDS Technical Architecture

**Figure 1:**
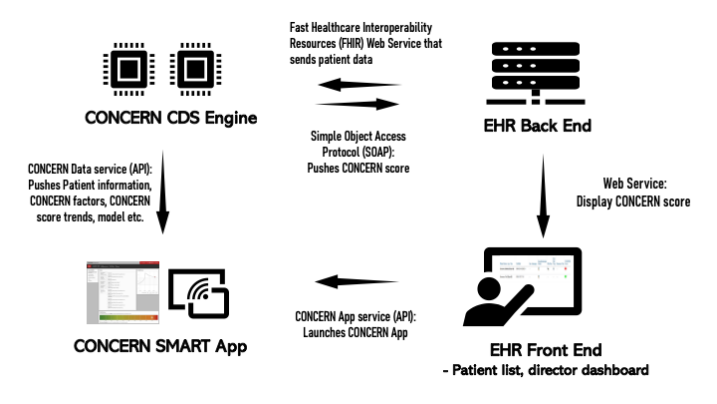
Technical architecture of the COmmunicating Narrative Concerns Entered by Registered Nurses (CONCERN) clinical decision support (CDS) app. API: application programming interface; EHR: electronic health record.

The CONCERN CDS app currently runs outside of the EHR (Figure 1). The process is as follows: (1) Fast Healthcare Interoperability Resources (FHIR) webservices (a combination of FHIR and Epic web services are being used at one site) pull data from the EHR backend into the CONCERN CDS engine. (2) The patients’ demographics, clinical notes, and app logging information are retrieved, and the CONCERN score is computed in the engine and pushed back to the EHR backend using a Simple Object Access Protocol (SOAP) service. (3) Then, the CONCERN level, corresponding color, and description to CONCERN score are displayed to the clinicians in their EHR's patient list and to nursing directors in their EHR’s unit dashboard. (4) Double-clicking the CONCERN level icon in the EHR front end will seamlessly bring the user to the CONCERN web app, which provides detailed information about the CONCERN model, including factors that contribute to each patient’s specific CONCERN level and CONCERN level trendline over 72 hours. Users can click on a specific factor and that data from the patient’s chart will be readily available. Given that the control and intervention groups of this study include inpatient acute and intensive care units, the color indicators are integrated into the existing EHR patient list and will only be visible for inpatients in these units.

## Methods

### CONCERN Intervention Trial Design

**Figure 2:**
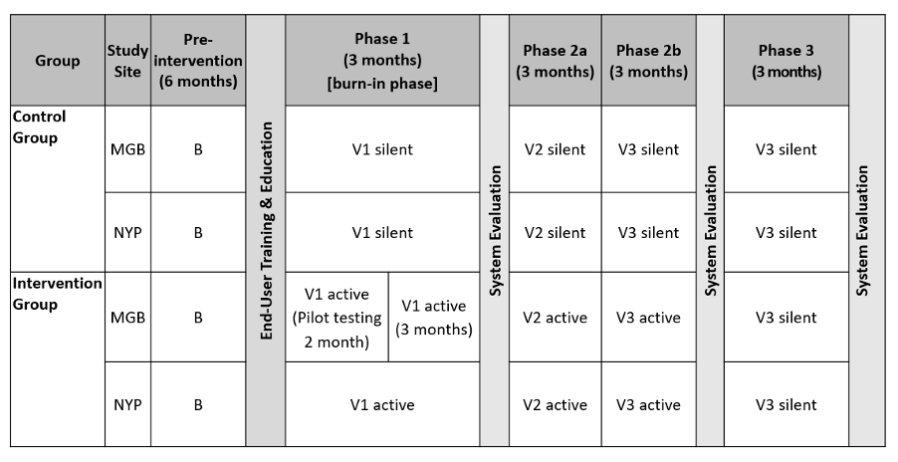
Cluster randomized pragmatic trial design with multiple time-series intervention. B: Baseline data; MGB: Mass General Brigham; NYP: New York Presbyterian; Silent: CONCERN CDS will function but will not display to clinician; Active: CONCERN CDS will display to clinician; V1: version 1, refined on the basis of continuous monitoring of data; V2: version 2, refined on the basis of continuous monitoring of data; V3: version 3, refined on the basis of continuous monitoring of data.

The CONCERN intervention trial design will be a cluster randomized pragmatic clinical trial with a multiple time-series intervention [14] (Figure 2). The trial has received institution review board approval at Columbia University and Mass General Brigham. A multiple time-series intervention is used to assess the impact of the CONCERN CDS system because it allows for periodic evaluation and model optimization (ie, we can refine our models on the basis of continuous data monitoring). Randomization will occur at the cluster level; we define the cluster as the clinical unit (ie, clinical ward) that the patients in our trial are admitted to. There will be a total of 86 intervention and control units across all sites, with the intervention and control groups randomly assigned using a random number generator. Study units include nonspecialty acute and intensive units. Randomization does not occur at the individual patient or clinician level in order to mitigate potential cross-over between control and intervention groups owing to clinician movement throughout the hospital. Baseline data will be collected prior to the intervention. Silent release mode (no CDS viewable to the end user) will be used in nonequivalent control units and as a postintervention unit control to evaluate whether notifying clinicians can decrease rates of negative clinical outcomes. Silent mode is a functionality that allows the CDS logic to actively run within the system and log its activity for later analysis, but not display any information or alerts to the user.

The primary outcomes are in-hospital mortality and length of stay; and secondary outcomes are cardiac arrest, unanticipated ICU transfers, and 30-day hospital readmission rates.

Three phases will be conducted through a 1-year period, and CONCERN CDS will be updated between each phase on the basis of the latest findings. Phase 1, the “burn-in” phase, will evaluate adoption of, and adaptation to, our algorithm. In the case of the Mass General Brigham site, the burn-in phase will be separated into two processes: pilot testing and trial testing. Phase 2 will activate the optimized versions 2 and 3 of the CONCERN CDS, which will be optimized based on experience from the burn-in phase 1. In the final phase, phase 3, the system will be set to silent release mode where no CONCERN levels are displayed within the patient list.

### CONCERN Intervention

**Figure 3:**
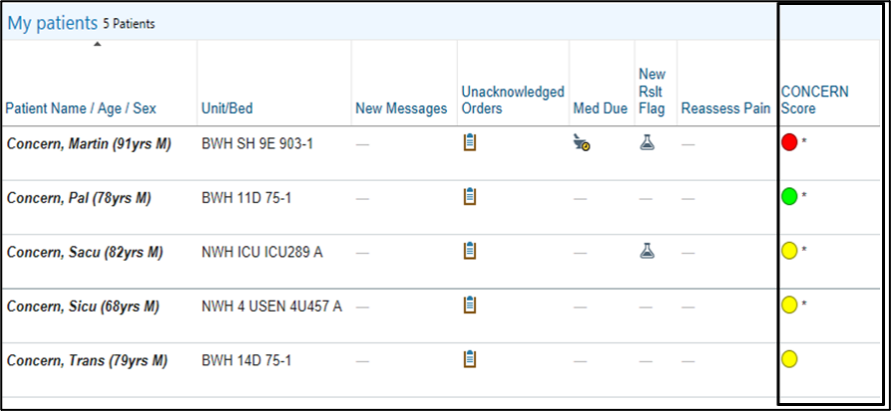
COmmunicating Narrative Concerns Entered by Registered Nurses (CONCERN) Score column in Mass General Brigham’s Epic patient list. Epic screen shot used with approval by 2021 Epic Systems Corporation. © 2021 Epic Systems Corporation.

Nurses and physicians who work on intervention units will have the CONCERN Score column integrated into their EHR patient list. The color-coded CONCERN score icon (red, yellow, green, and gray), which indicates the level of patient risk for deterioration, will be displayed in this column (Figure 3). The control units will not contain the CONCERN score column. Even if control group participants manage to manually add the CONCERN score column to their patient list view by themselves, only gray color (nonscores) will be displayed since their units and patients are excluded from the model.

### Clinician Training

A training period will be conducted with nurse and physician end users from the intervention units prior to implementation to promote effective use of the CONCERN CDS app. The training plan was established with physician and nursing leaders who participated in user-centered design, focus group interviews, and simulation sessions. The training curriculum will last approximately 30 minutes and will be executed in groups or individually in consideration of each clinician’s schedule. In addition, educational materials such as informative posters, pocket reference cards, and CONCERN tip sheets will be distributed on intervention units before and throughout the trial.

### Study Setting

The CONCERN CDS system will be integrated into the EHR at New York Presbyterian’s Columbia University Medical Center (NYP-CUMC) and the Allen Hospital (NYP-Allen), Mass General Brigham’s Brigham and Women’s Hospital (MGB-BWH), and Newton-Wellesley Hospital (MGB-NWH). Our system will be implemented in 21 intervention and control units (721 beds) at NYP-CUMC, 5 intervention and control units (147 beds) at NYP-Allen, 55 intervention and control units (663 beds) at MGB-BWH, and 5 intervention and control units (153 beds) at MGB-NWH, yielding a total of 86 intervention and control units across all sites. Clinical unit room and bed counts are known to fluctuate slightly as a result of continuous updates in hospital operations, including in response to the COVID-19 pandemic. Therefore, the counts listed are subject to change.

### Study Participants

All patients over 18 years of age admitted to one of our 86 study units will be enrolled in the trial. Hospice and palliative care patients will be excluded. All nurses and physicians at our study sites will be eligible to participate in a usability survey. All clinicians are expected to be above the age of 21 years of age. Minors will be excluded from participating in the study.

### Data Collection and Analysis

#### Qualitative Evaluation

The individual and group interviews will be conducted with nurses and physicians who have used the CONCERN CDS app. They will mainly focus on the following: (1) assessing perceived understandability of the CONCERN CDS app, (2) evaluating perceived technical competence with the CONCERN CDS app, and (3) gauging trust in the CONCERN CDS app (as influenced by understandability and technical competence). Participation will be voluntary, and all recruitment materials—including emails, flyers, and information sheets—will reflect that fact. The interviews will be conducted and recorded using Zoom (a Health Insurance Portability and Accountability Act [HIPAA]–compliant Zoom account will be used for all interviews) or in person, transcribed using a HIPAA-compliant professional service, and coded using NVivo software.

#### Quantitative Evaluation

Quantitative CONCERN intervention trial data collection and analysis includes the collection of pre- and postintervention data during the 6 months before and 12 months after implementation. Prior to conducting hypotheses tests, descriptive statistics will be used to describe outcome variables and key confounding variables. We propose a generalized linear mixed model to examine the impact of the CONCERN system on each of the two primary outcomes: in-hospital mortality and length of stay. This model is used to deal with combined data from multiple sites and can account for changes over time. It also allows different baselines and trajectories to account for the contrasting ICU and non-ICU settings and can include both patient-level and unit-level covariates. We will include a variable for closed versus open units in our analysis. We will estimate statistical power for the comparison of mortality rates between the intervention and nonintervention periods and between silent- and active-model periods.

All power calculations will be based on 2-sided tests with Cronbach α=.05. Using hospitalized patients’ statistics, we expect at least 2000 total admissions per month (ranging from 38 to 270 admissions in different units) with a mean length of stay of approximately 6 days. Each intervention period (pre- and postintervention periods) will be 6 months; therefore, we expect approximately 12,000 patients in each period. For length of stay outcome analysis, we will have at least 80% statistical power to detect a relative difference of 2% in the length of stay. Based on hospitalized patients’ statistics, mean mortality rates of 37.5 (range 11.9 to 48.8) deaths per 10,000 inpatient days were obtained. Using a conservative number of total 50,000 inpatient days in each intervention period, we will have at least 80% statistical power to detect a relative difference of risk ratio of 0.76 in mortality rates.

There is no consensus regarding the best method for analyzing length of stay. Length of stay has been analyzed using both Poisson models (or negative binomial [NB] models or other related models such as Zero-truncated Poisson models) and survival models (such as Cox proportional hazard models) [16]. Both approaches will be applied to the length of stay, and the better predictive performing model, measured by the average squared error (ASE) for individuals from a testing data set, will be retained. Secondary outcomes include 30-day hospital readmission, cardiac arrest, and unanticipated transfers to the ICU, as well as analytics of CONCERN system log-files for clinician usage metrics.

## Results

This trial has been approved by the institutional review boards at Columbia University Medical Center (protocol AAAR1389) and Mass General Brigham (IRB protocol 2015P002472). Data collection and analysis are expected to conclude by August 2022. The CONCERN CDS system is expected to have a positive impact on patient health outcomes (ie, reduced mortality and shorter hospital stays).

## Discussion

### Expected Findings

The CONCERN CDS leverages our predictive algorithm to determine and assign each patient a “CONCERN Level” (red, yellow, or green), representing a patient’s risk level for deterioration. The CONCERN CDS has the potential to impact yellow-coded patients most significantly, as greater care team awareness, increased surveillance, and early interventions can help prevent these vulnerable patients from deteriorating. Generally speaking, CONCERN will be less impactful on patients who receive a red CONCERN level because they are likely actively deteriorating to such an extent that clinicians will already be aware of those declining health statuses.

### Reduce CDS Alarm Fatigue

CDS alarm fatigue has been reported as a threat to patient safety [17]. We have explored this phenomenon in user-centered design and focus group interviews and found that clinicians do not want interruptive alarms, as these contribute significantly to alarm fatigue. Therefore, the CONCERN CDS app was integrated into the existing EHR patient list function and was designed to only display colored circle icons to easily communicate the degree of deterioration risk without interrupting clinician workflow. In addition, an asterisk (*) will appear next to the CONCERN level icon any time the score’s color has recently changed (eg, a score that has changed from green to yellow, red to green, etc, within the past hour).

We also addressed CONCERN CDS scoring sensitivity in our algorithm development. If the scores are too sensitive, and the system designates too many patients as at above-average risk, over time they could cause users to perceive the tool as meaningless, especially in the ICU. The distributions of risk scores present at any given time are therefore controlled by our algorithm, limiting the number of patients who receive yellow and red scores to simply 10% and 2%, respectively. Score sensitivity is further controlled on the basis of unit type (ICU versus non-ICU), as ICU patients at baseline tend to be in more critical health states and require more attention and extreme treatment measures than non-ICU patients.

### Risks and Discomforts

Because the core function of the CONCERN CDS app is to aid clinicians recognizing and taking measures to quickly and effectively treat patients at risk for deterioration, there are no anticipated physical risks in the study. Moreover, the CONCERN CDS will initially be deployed in “silent” mode, allowing for adequate evaluation and validation that it is functioning properly before it is activated for clinicians providing patient care. The CONCERN CDS app will be monitored closely with manual and automated review of log-files to ensure it is suitable for patient care. If any critical issue with the system should arise, which negatively impacts or impedes patient care, it will be immediately turned off, while diagnostic measures, such as retracing the software development lifecycle steps, are to be performed and solutions would be found. Then, only after thorough testing of the updated system, specifications, and fixes to the software reliably demonstrate that the solutions have addressed those issues, will the system be reactivated.

### Integration Within Workflow

Implementation of the CONCERN CDS app is focused on integrating the tool within the users’ existing workflow as seamlessly as possible. To best complement established clinical environments and cultures, the CONCERN implementation training strategy was constructed with physician and nursing leaders. The first step of this strategy is selecting champions among the professional development managers and nurses in charge from intervention units. The champions play an integral role in ensuring that integration of the CONCERN CDS app into their units’ workflows is carried out harmoniously and in facilitating communication channels between the tool’s end users and study team. Champions will also be trained as CONCERN “superusers,” who in turn will train and support clinicians on the processes and rationale for using the app through all facets of the pre-, mid-, and postimplementation periods. Similarly, the CONCERN team will provide champions with educational material (eg, posters and pocket cards), maintain constant contact, and remain easily accessible to assist them in these training efforts whenever and however needed.

### Study Limitations

As with all EWS models, clinical outcomes may underrepresent the impact of an EWS intervention on “at-risk” patients who receive timely and successful interventions and therefore do not experience a negative outcome. Additionally, the impact of clinician expertise and team coherence on patient outcomes has been considered and should be studied. However, quantifying information about the quality of the nurse and physician relationship into actionable CDS for predictive validity is not currently feasible and is beyond the scope of this study. The interplay of personality, temperament, and other factors in addition to work history would be pertinent for such an analysis. SCR’s work at MGB and NYP [5,6,18,19] validated that care teams consistently leverage verbal conversations for decision-making while referencing CDS and other EHR information. Therefore, the extent to which expertise of one care team member or trainee has influenced a decision and the team’s dynamic is either not consistently recorded in the EHR, or if recorded is likely not documented in real time [19]. Thus, it is not practicable to perform real-time predictive analytics of these types of care team dynamics. However, by designing the CONCERN CDS algorithm to utilize data over 12 hours for hourly calculations, we are able to capture and use any documentation that is delayed and is not in real time. Another potential limitation is that the CONCERN system might cause clinicians to overlook patients with a green CONCERN level because of perceived low risks. Nonetheless, during training, clinicians are encouraged to use the tool as an additional feature that helps them with CDS rather than a definitive tool to guide their decisions. We shall obtain a better understanding of the effect of the CONCERN CDS and its impact on clinician behaviors during outcome evaluation.

### Conclusions

Our study defined a new source of predictive data by analyzing the types and frequencies of nursing documentation indicative of nurses’ developing concerns about their patients, and subsequent increased surveillance, to build an early warning score system—the CONCERN CDS system. The CONCERN CDS system will be released and evaluated in 2 different health care systems through a multiple time-series trial protocol that consists of 3 phases. The impact on health outcomes and the usability of the CONCERN CDS by the end users will be evaluated through a mixed methods (quantitative and qualitative) approach. We expect the CONCERN CDS will increase team-based situational awareness, shared understanding of the patient situation, and timely recognition of patients predicted to be at risk for deterioration to influence rapid intervention that prevents mortality and associated harm.

## Abbreviations

ASE: average squared error
CDS: clinical decision support
CONCERN: COmmunicating Narrative Concerns Entered by Registered Nurses
EHR: electronic health record
EWS: early warning system
FHIR: Fast Healthcare Interoperability Resources
HIPAA: Health Insurance Portability and Accountability Act
ICU: intensive care unit
MEWS: Modified Early Warning Score
MGB: Mass General Brigham’s
MGB-BWH: Mass General Brigham’s Brigham and Women’s Hospital
MGB-NWH: Mass General Brigham's Newton-Wellesley Hospital
NB: negative binomial
NLP: natural language processing
NYP-CUMC: New York Presbyterian’s Columbia University Medical Center
PRN: pro re nata
SOAP: Simple Object Access Protocol
